# The association between acute nutritional changes and prognosis in ischemic stroke patients

**DOI:** 10.3389/fnut.2026.1803930

**Published:** 2026-04-15

**Authors:** Zhuangzhuang Jiang, Meiyun Zhang, Jingjing Li, Xiaorong Lu, Xiaolan Wu, Yuan Fang, Liang Wang, Dongjuan Xu

**Affiliations:** 1 Department of Neurology, Affiliated Dongyang Hospital of Wenzhou Medical University, Dongyang, China; 2 Department of Patient Services Management, Affiliated Dongyang Hospital of Wenzhou Medical University, Dongyang, China; 3 Department of Clinical Laboratory, Affiliated Dongyang Hospital of Wenzhou Medical University, Dongyang, China

**Keywords:** functional outcomes, ischemic stroke, nutritional deterioration, nutritional status changes, Prognostic Nutritional Index

## Abstract

**Background:**

Nutritional status during the acute phase of ischemic stroke can change dynamically and may influence patient outcomes. However, the impact of short-term nutritional changes on prognosis remains unclear. This study aimed to investigate the association between acute-phase nutritional changes and 3-month functional outcomes, and to identify factors contributing to nutritional deterioration.

**Methods:**

A prospective–retrospective cohort study was conducted including 1,445 patients with acute ischemic stroke admitted within 48 h of onset. Nutritional status was assessed using the Prognostic Nutritional Index (PNI) within 24 h of admission (PNI1) and on day 5 ± 1 of admission (PNI2). ΔPNI was calculated as PNI2 minus PNI1. Functional outcomes at 3 months were evaluated using the modified Rankin Scale (mRS), with mRS ≥3 defined as poor outcome. Logistic regression and restricted cubic spline analyses were performed to examine the association between ΔPNI and outcomes. Subgroup analyses explored potential effect modifiers, and linear regression identified determinants of nutritional changes.

**Results:**

PNI2 was significantly lower than PNI1 (45.75 vs. 46.70, *p* < 0.001). An increase in ΔPNI was independently associated with a decreased likelihood of poor 3-month outcomes (Model 4: OR per 5-unit increase = 0.744, 95% CI: 0.579–0.957, *p* = 0.021). Patients in the nutritional improvement group showed significantly better outcomes than those in the deterioration group (ΔPNI_ ± 1.5 OR: 0.559, 95% CI: 0.330–0.946, *p* = 0.030; ΔPNI/PNI1_ ± 3% OR: 0.556, 95% CI: 0.334–0.928, *p* = 0.025). Subgroup analyses indicated stronger associations in patients aged <65 years, males, those with large-artery atherosclerosis, mild stroke, or receiving intravenous thrombolysis. Older age, low baseline PNI, low body mass index, and nasogastric tube feeding were significant risk factors for nutritional deterioration.

**Conclusion:**

Declines in nutritional status during the acute phase of ischemic stroke are independently associated with an increased risk of poor 3-month functional outcomes, particularly in specific patient subgroups. Patients with low baseline energy reserves, older age, or requiring nasogastric tube feeding are at higher risk of nutritional deterioration.

## Introduction

According to the Global Burden of Disease 2021 estimates, stroke remains the second leading cause of death among non-communicable diseases worldwide, accounting for nearly 7 million deaths annually and over 160 million disability-adjusted life years lost (1, 2). Ischemic stroke is the predominant subtype, with 7.804 million new cases reported in 2021, representing approximately 65.3% of all incident strokes (3). In the same year, the global prevalence of ischemic stroke reached 69.945 million cases, more than doubling from 34.668 million cases in 1990 (1). Notably, 59% of prevalent cases occurred in individuals under the age of 70, suggesting that a substantial proportion of the working-age population may face long-term disability, reliance on family care, and ongoing medical interventions (2).

Nutrition is closely associated with ischemic stroke. Individuals with a well-balanced diet and good nutritional status have a markedly lower risk of incident ischemic stroke (4, 5). Moreover, nutritional status during the acute phase of ischemic stroke is strongly linked to patient outcomes. Higher serum albumin levels, a widely used marker of nutritional status, are associated with better prognosis (6, 7). Studies employing nutritional assessment tools such as Geriatric Nutritional Risk Index (GNRI), Controlling Nutritional Status (CONUT) score, and Prognostic Nutritional Index (PNI) have further shown that better nutritional status reliably predicts improved functional recovery, as well as lower rates of complications and mortality (8, 9). However, most previous studies have focused on the relationship between baseline nutritional status and outcomes, largely overlooking dynamic changes in nutritional status (8–10). Consequently, the changes in nutritional status during the acute phase of ischemic stroke remain poorly understood, and it is unclear whether these fluctuations influence stroke prognosis.

The aim of this study was to examine the relationship between changes in nutritional status during the acute phase of ischemic stroke and 3-month outcomes. Additionally, the study sought to identify the risk factors contributing to nutritional changes during this period.

## Materials and methods

### Study cohort

This prospective–retrospective cohort study was conducted at Dongyang People’s Hospital. The study was approved by the Ethics Committee of Dongyang People’s Hospital and conducted in accordance with the Declaration of Helsinki. Written informed consent was obtained from all participants or their legal guardians. Hospitalized patients in the Department of Neurology were eligible if they met the following criteria: (1) confirmed diagnosis of ischemic stroke according to the 2023 Chinese Guidelines for the Diagnosis and Treatment of Acute Ischemic Stroke (11); (2) age >18 years; and (3) admission within 48 h of symptom onset. Exclusion criteria were: (1) hospital stay <48 h; (2) presence of acute infections during hospitalization; (3) baseline modified Rankin Scale (mRS) score ≥3 prior to stroke onset; (4) missing clinical data or loss to follow-up. None of the study participants received intravenous albumin or other nutrition-related therapies.

### Nutritional assessment selection

A variety of tools are currently available for assessing nutritional status. Since this study aimed to investigate the relationship between short-term changes in nutritional status during the acute phase of ischemic stroke and clinical outcomes, we required an assessment tool capable of detecting rapid nutritional changes. The Subjective Global Assessment and Mini Nutritional Assessment rely on measurements such as subcutaneous fat thickness and limb circumference (12, 13), which are unlikely to change significantly in the short term. In the Nutritional Risk Screening 2002 (14), the nutritional impairment component primarily reflects changes in body mass index (BMI) and dietary intake over several weeks, while the disease severity component focuses on acute illness; moreover, the age score (≥70 years) is fixed, making it unsuitable for evaluating short-term nutritional changes in this study. The CONUT score incorporates serum cholesterol levels (15), but since ischemic stroke patients are routinely treated with statins or other lipid-lowering agents upon admission, it may not accurately reflect their true nutritional status. Therefore, we selected the PNI as the most appropriate tool for this study. PNI was calculated as serum albumin (g/L) + 5 × total lymphocyte count (×10^9^/L) (16). Both serum albumin and lymphocyte count change rapidly and can sensitively reflect short-term variations in nutritional status. PNI measured within 24 h of admission was defined as PNI1, and PNI measured on day 5 ± 1 after admission was defined as PNI2. The change in nutritional status was expressed as ΔPNI = PNI2 − PNI1.

### Data collection

We gathered a comprehensive set of baseline characteristics, including demographic data, vascular risk factors, comorbidities and laboratory results. Hypertension was identified by the prior use of antihypertensive medications. Diabetes mellitus was defined as the use of glucose-lowering medications or hemoglobin A1c ≥6.5%. Ischemic heart disease and atrial fibrillation were considered present if there was clear medical documentation or a confirmed diagnosis at discharge. Current cigarette smoking and alcohol consumption of 15 g or more per day in the past year were defined accordingly. Reperfusion therapy included intravenous thrombolysis, emergent endovascular intervention, or bridging therapy. Bridging therapy was grouped with emergent endovascular intervention for analytical purposes due to the limited number of cases. Patients with a Kubota water drinking test score below 3 were considered to have normal swallowing function and received oral feeding, whereas those with a score of 3 or higher or with impaired consciousness were classified as having dysphagia and received nasogastric tube feeding (17). Nutrition modality was classified as oral or nasogastric tube, with tube feeding administered for more than 48 h defined as tube-fed nutrition. Neurological status was assessed on admission using the National Institutes of Health Stroke Scale (NIHSS) by experienced neurologists (18). Stroke etiology was classified according to the Trial of Org 10172 in Acute Stroke Treatment (TOAST) criteria based on medical history and admission findings (19). At 3 months ±1 week after stroke onset, outcomes were evaluated via outpatient visits or telephone follow-up using the modified Rankin Scale (mRS) (20), with mRS ≥3 defined as a poor outcome, indicating moderate-to-severe disability or death after stroke.

### Sample size calculation

The sample size for this study was determined based on a retrospective study conducted from January to December 2024, which reported an incidence of positive outcome events (mRS ≥3 at 3 months after stroke onset) of 14.2% (132/930). A total of 16 independent variables (PNI1, ΔPNI, age, gender, BMI, hypertension, diabetes, baseline NIHSS score, nutrition modality, TOAST classification (4 categories), reperfusion therapy, TG, TC, LDL, HDL, and glycated hemoglobin) were planned for inclusion in the final analysis model, based on the retrospective results and previous literature. Following the 10-events-per-variable (EPV) principle for binary logistic regression (21), a minimum of 190 positive outcome events (19 variables × 10 events, including dummy variables) was required. Dividing the required number of events by the expected event rate (14.2%) yielded a total sample size of 1,338 patients. After accounting for the 930 cases already included in the retrospective study, an additional 408 patients were needed for the prospective cohort. Based on the retrospective data, a 20% patient dropout rate and a 20% loss of repeated PNI measurements were anticipated, the final prospective sample size was calculated as 408 × 1/(1–0.20) × 1/(1–0.20) ≈ 638. Accordingly, at least 638 patients with acute ischemic stroke will be enrolled in the prospective cohort.

### Statistical analysis

Continuous variables were presented as medians with interquartile ranges (IQRs), and categorical variables as counts and percentages. Comparisons of continuous variables were performed using the Mann–Whitney *U* test for two independent groups, the Kruskal–Wallis *H* test for three independent groups, and the Wilcoxon signed-rank test for paired samples. Differences in categorical variables were assessed using the chi-square (*χ*^2^) test. To examine the potential independent association between ΔPNI and 3-month outcomes after ischemic stroke, four logistic regression models were constructed. ΔPNI was included in the regression models both as a continuous variable and as a categorical variable. For the categorical analyses, three classification methods were applied: (1) tertiles of ΔPNI (T1: lowest third, T2: middle third, T3: highest third); (2) centered around 0 with a ± 1.5-point range (deterioration group: ΔPNI <−1.5; stable group: –1.5 ≤ΔPNI ≤1.5; improvement group: ΔPNI >1.5); and (3) relative change from PNI1 (ΔPNI/PNI1) (deterioration group: <−3%; stable group: −3 to 3%; improvement group: >3%). Candidate covariates for adjustment were selected based on intergroup comparisons and previous literature. Trend tests were performed for all categorical analyses. Restricted cubic spline (RCS) analysis was used to explore potential linear and non-linear associations between ΔPNI and prognosis. Subgroup analyses were conducted to investigate potential moderators of the ΔPNI–prognosis relationship. To identify factors associated with PNI2, covariates were pre-specified based on clinical relevance and prior evidence from the literature and grouped as follows: demographics (age, sex), baseline nutritional status (PNI1, BMI), stroke severity (NIHSS score), metabolic disorders (diabetes mellitus, hyperlipidemia), comorbidities (hypertension, ischemic heart disease, history of stroke/transient ischemic attack), lifestyle factors (excessive alcohol consumption), and nutritional support modality (22–24). All selected variables were simultaneously entered into the multivariable linear regression model using the enter method. Statistical significance was defined as a two-sided *p* < 0.05. All analyses were performed using SPSS version 26.0, GraphPad Prism version 9.0, and R version 4.1.1.

## Results

### Baseline characteristics and PNI changes over time

**Figure 1:**
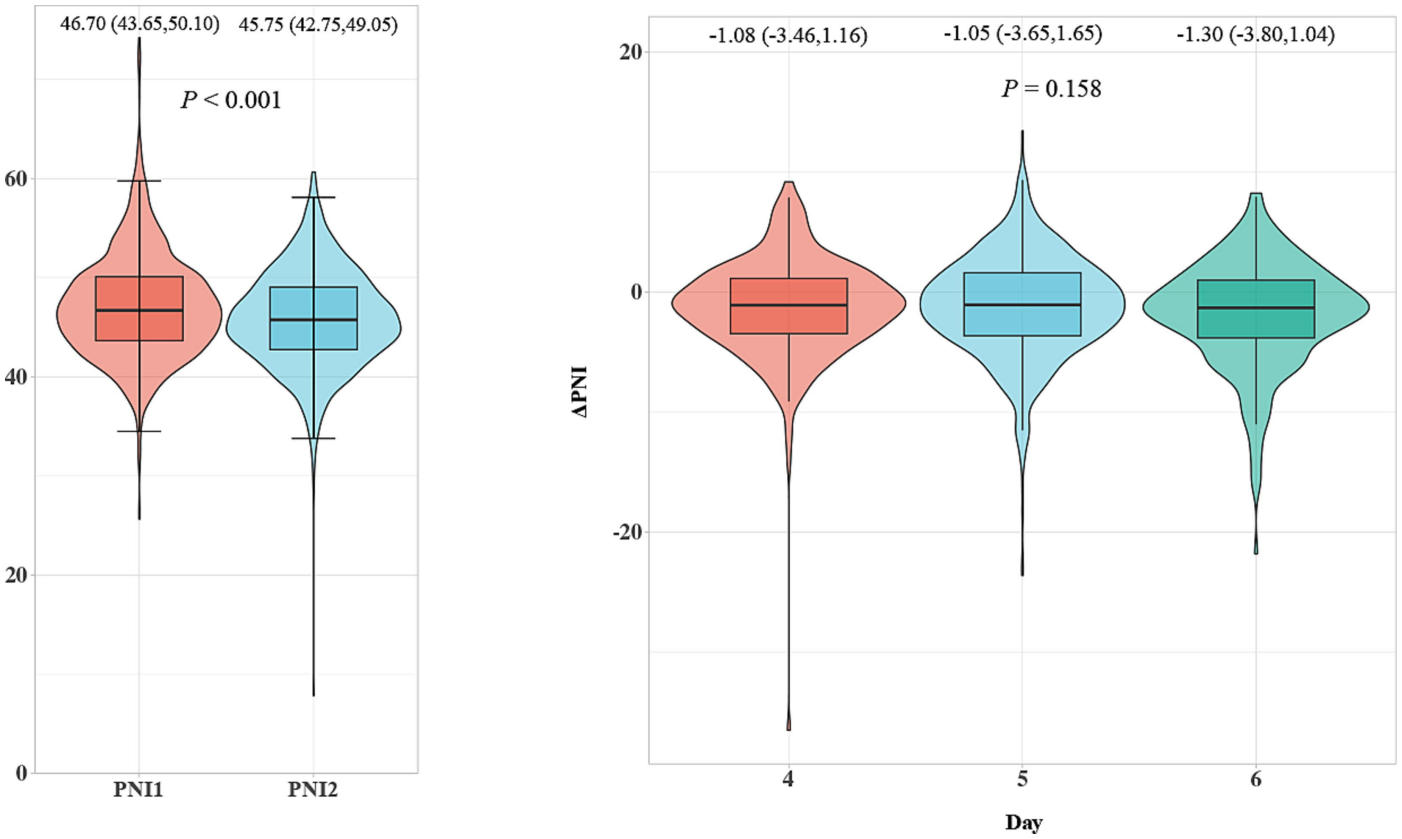
Temporal changes in PNI during the acute phase of ischemic stroke. PNI decreased significantly from PNI1 to PNI2 (*p* < 0.001, Wilcoxon signed-rank test), whereas ΔPNI showed no significant difference across the three measurement days (*p* = 0.158, Kruskal–Wallis *H* test). PNI, Prognostic Nutritional Index.

Based on the sample size calculation, we retrospectively collected ischemic stroke cases from January to December 2024 and prospectively collected cases from January to June 2025 that met the study inclusion criteria. A total of 1,445 patients were included, with a median age of 69.0 (58.0, 77.0) years, and 932 (64.5%) were male. Among them, 208 patients (14.4%) had a 3-month mRS score ≥3. Table 1 presents the baseline characteristics stratified by mRS (<3 vs. ≥3). Patients with poor functional outcomes were older [73.00 (59.75, 80.00) vs. 68.00 (58.00, 77.00), *p* < 0.001] and had a lower proportion of males (56.7% vs. 65.8%, *p* = 0.011). Baseline stroke severity was substantially higher in the poor-outcome group, as reflected by elevated NIHSS scores [5.00 (3.00, 9.00) vs. 1.00 (1.00, 3.00), *p* < 0.001]. TOAST subtype distribution also differed significantly, with higher frequencies of large-artery atherosclerosis (31.7% vs. 22.2%) and cardioembolism (10.6% vs. 7.7%) in patients with mRS ≥3 (overall *p* < 0.001). Regarding treatments, patients with poor outcomes were more likely to receive reperfusion therapy (27.9% vs. 11.6%, *p* < 0.001) and to require nasogastric tube feeding (20.2% vs. 4.5%, *p* < 0.001). Several laboratory parameters differed between groups. Patients with poor outcomes had lower TG levels [1.05 (0.75, 1.53) vs. 1.19 (0.87, 1.67), *p* = 0.001] and higher LDL [2.49 (1.66, 3.06) vs. 2.12 (1.50, 2.85), *p* = 0.002] and TC levels [4.03 (3.11, 4.79) vs. 3.77 (3.03, 4.54), *p* = 0.011]. Baseline PNI (PNI1) did not differ significantly between outcome groups [46.48 (43.31, 49.88) vs. 46.75 (43.75, 50.15), *p* = 0.531]. In contrast, follow-up (PNI2) was significantly lower in patients with poor outcomes compared with those with favorable outcomes [44.58 (41.55, 47.30) vs. 46.00 (43.00, 49.20), *p* < 0.001], and ΔPNI was more negative in the poor-outcome group [−1.83 (−4.25, 0.08) vs. −0.95 (−3.55, 1.55), *p* < 0.001]. As shown in Figure 1, PNI2 showed a significant decline compared with PNI1 [46.70 (43.65, 50.10) vs. 45.75 (42.75, 49.05), *p* < 0.001], whereas ΔPNI did not differ significantly across the three measurement days [−1.08 (−3.46, 1.16) vs. −1.05 (−3.65, 1.65) vs. −1.30 (−3.80, 1.04), *p* = 0.158].

**Table 1:** Baseline patient characteristics stratified by 3-month functional outcome.

Variables	All patients (*n* = 1,445)	MRS ≥3 (*n* = 208)	MRS<3 (*n* = 1,237)	*p-*value
Demographic data				
Age (years), median (IQR)	69.00 (58.00, 77.00)	73.00 (59.75, 80.00)	68.00 (58.00, 77.00)	<0.001
Sex, male, *n* (%)	932 (64.5%)	118 (56.7%)	814 (65.8%)	0.011
BMI, median (IQR)	24.00 (21.80, 26.30)	23.35 (21.40, 26.30)	24.00 (22.00, 26.30)	0.080
Vascular risk factors, *n* (%)				
Hypertension	1,143 (79.1%)	167 (80.3%)	976 (78.9%)	0.649
Diabetes mellitus	469 (32.5%)	73 (35.1%)	396 (32.0%)	0.380
Ischemic heart disease	204 (14.1%)	30 (14.4%)	174 (14.1%)	0.891
Atrial fibrillation	162 (11.2%)	27 (13.0%)	135 (10.9%)	0.382
History of stroke/TIA	227 (15.7%)	33 (15.9%)	194 (15.7%)	0.947
Current smoke	418 (28.9%)	55 (26.4%)	363 (29.3%)	0.393
Excess alcohol consumption	288 (19.9%)	39 (18.8%)	249 (20.1%)	0.645
Treatment characteristics, *n* (%)				
Pre-stroke antithrombotic use	267 (18.5%)	35 (16.8%)	232 (18.8%)	0.507
Pre-stroke lipid-lowering therapy	249 (17.2%)	31 (14.9%)	218 (17.6%)	0.337
Acute treatment modality				<0.001
No reperfusion therapy	1,243 (86.0%)	150 (72.1%)	1,093 (88.4%)	
Intravenous thrombolysis	149 (10.3%)	48 (23.1%)	101 (8.2%)	
Endovascular treatment	53 (3.7%)	10 (4.8%)	43 (3.5%)	
Nutritional support				<0.001
Oral feeding	1,347 (93.2%)	166 (79.8%)	1,181 (95.5%)	
Nasogastric tube feeding	98 (6.8%)	42 (20.2%)	56 (4.5%)	
Baseline data				
Admission NIHSS score, median (IQR)	2.00 (1.00, 3.00)	5.00 (3.00, 9.00)	1.00 (1.00, 3.00)	<0.001
Admission NIHSS score, *n* (%)				<0.001
0–3	1,146 (79.3%)	78 (37.5%)	1,068 (86.3%)	
4–6	165 (11.4%)	50 (24.0%)	115 (9.3%)	
>6	134 (9.3%)	80 (38.5%)	54 (4.4%)	
TOAST classification, *n* (%)				<0.001
Large-artery atherosclerosis	340 (23.5%)	66 (31.7%)	274 (22.2%)	
Cardioembolism	117 (8.1%)	22 (10.6%)	95 (7.7%)	
Small-vessel occlusion	728 (50.4%)	102 (49.0%)	626 (50.6%)	
Other/Undetermined	260 (18.0%)	18 (8.7%)	242 (19.6%)	
Laboratory data, median (IQR)				
Hemoglobin (g/L)	137.00 (125.00, 149.00)	138.00 (127.00, 150.00)	137.00 (125.00, 148.00)	0.130
TG (mmol/L)	1.18 (0.85, 1.66)	1.05 (0.75, 1.53)	1.19 (0.87, 1.67)	0.001
LDL (mmol/L)	2.17 (1.52, 2.88)	2.49 (1.66, 3.06)	2.12 (1.50, 2.85)	0.002
HDL (mmol/L)	1.03 (0.88, 1.23)	1.05 (0.91, 1.24)	1.02 (0.88, 1.22)	0.143
TC (mmol/L)	3.83 (3.03, 4.59)	4.03 (3.11, 4.79)	3.77 (3.03, 4.54)	0.011
Hemoglobin A1c (%)	6.00 (5.70, 6.70)	5.95 (5.70, 6.70)	6.00 (5.70, 6.70)	0.844
PNI1	46.70 (43.65, 50.10)	46.48 (43.31, 49.88)	46.75 (43.75, 50.15)	0.531
PNI2	45.75 (42.75, 49.05)	44.58 (41.55, 47.30)	46.00 (43.00, 49.20)	<0.001
ΔPNI	−1.15 (−3.65, 1.40)	−1.83 (−4.25, 0.08)	−0.95 (−3.55, 1.55)	<0.001

BMI, body mass index; mRS, modified Rankin Scale; TIA, transient ischemic attack; NIHSS, National Institutes of Health Stroke Scale; TOAST, Trial of Org 10172 in Acute Stroke Treatment; TG, triglyceride; LDL, low-density lipoprotein cholesterol; HDL, high-density lipoprotein cholesterol; TC, total cholesterol; PNI, Prognostic Nutritional Index; IQR, interquartile range.

### Association between ΔPNI and 3-month outcomes

As shown in Table 2, higher ΔPNI was consistently associated with a lower risk of poor 3-month outcomes. When analyzed as a continuous variable, each 5-unit increase in ΔPNI was independently protective across all four models (adjusted ORs 0.699–0.756, all *p* < 0.05). For the tertile classification, only the highest ΔPNI tertile (T3) was associated with reduced odds of poor outcome (Model 4: OR = 0.478, 95% CI 0.286–0.799, *p* = 0.005), with significant linear trends across models (all *p* for trend ≤0.001). Using the ±1.5 grouping, the improvement group showed lower odds of poor outcome compared with the deterioration group (Model 4: OR = 0.559, 95% CI 0.330–0.946, *p* = 0.030), while the stable group showed no significant association. Trend tests remained significant (*p* for trend 0.007–<0.001). Similarly, for the relative change classification (ΔPNI/PNI1 ± 3%), only the improvement group was protective (Model 4: OR = 0.556, 95% CI 0.334–0.928, *p* = 0.025), with significant trends across all models (*p* for trend 0.005–<0.001).

**Table 2:** Association between PNI change in acute ischemic stroke and 3-month functional outcome.

ΔPNI	Model 1 OR (95% CI)	*p*	Model 2 OR (95% CI)	*p*	Model 3 OR (95% CI)	*p*	Model 4 OR (95% CI)	*p*
Per 5-unit increase	0.756 (0.642, 0.889)	<0.001	0.699 (0.565, 0.866)	0.001	0.750 (0.585, 0.963)	0.024	0.744 (0.579, 0.957)	0.021
ΔPNI_Tertile								
T1	ref		ref		ref		ref	
T2	0.809 (0.577, 1.134)	0.219	0.786 (0.544, 1.135)	0.199	0.874 (0.569, 1.343)	0.539	0.858 (0.557, 1.322)	0.486
T3	0.442 (0.301, 0.649)	<0.001	0.424 (0.275, 0.653)	<0.001	0.496 (0.299, 0.823)	0.007	0.478 (0.286, 0.799)	0.005
*p* for trend		<0.001		<0.001		0.001		0.001
ΔPNI_ ± 1.5								
Deterioration group	ref		ref		ref		ref	
Stable group	0.733 (0.522, 1.030)	0.074	0.742 (0.514, 1.072)	0.112	0.772 (0.504, 1.182)	0.234	0.754 (0.490, 1.159)	0.115
Improvement group	0.511 (0.340, 0.768)	0.001	0.509 (0.327, 0.791)	0.003	0.578 (0.344, 0.969)	0.038	0.559 (0.330, 0.946)	0.030
*p* for trend		<0.001		0.002		0.008		0.007
ΔPNI/PNI1_ ± 3%								
Deterioration group	ref		ref		ref		ref	
Stable group	0.706 (0.497, 1.004)	0.053	0.717 (0.493, 1.043)	0.082	0.766 (0.496, 1.182)	0.228	0.747 (0.481, 1.158)	0.192
Improvement group	0.501 (0.337, 0.744)	<0.001	0.497 (0.323, 0.763)	0.001	0.573 (0.346, 0.947)	0.030	0.556 (0.334, 0.928)	0.025
*p* for trend		<0.001		0.001		0.005		0.005

Model 1: Crude.

Model 2: Adjustment for age, gender, BMI, HPT, DM, PNI1.

Model 3: Model 2 along with NIHSS, TOAST classification, acute treatment modality, nutrition style.

Model 4: Model 3 along with LDL, HDL, TC, TG, HbA1c.

BMI, body mass index; HPT, hypertension; DM, diabetes mellitus; PNI, Prognostic Nutritional Index; NIHSS, National Institutes of Health Stroke Scale; TOAST, Trial of Org 10172 in Acute Stroke Treatment; LDL, low-density lipoprotein cholesterol; HDL, high-density lipoprotein cholesterol; TC, total cholesterol; TG, triglyceride; HbA1c, hemoglobin A1c.

### Restricted cubic spline analysis of ΔPNI and poor functional outcomes

**Figure 2:**
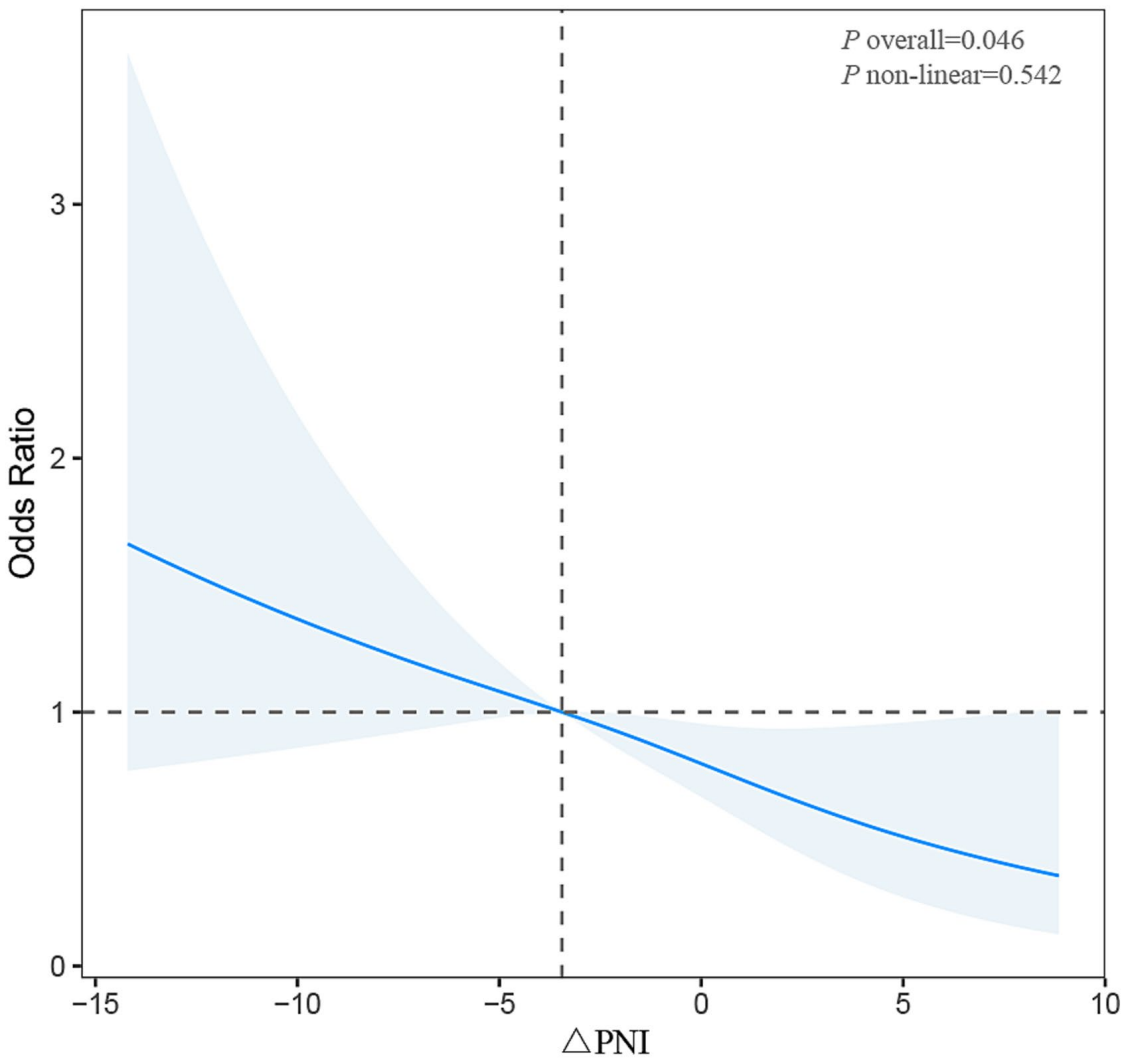
Restricted cubic spline showing a significant linear inverse association between ΔPNI and poor 3-month functional outcomes (mRS ≥3) (*p* overall = 0.046; *p* for nonlinearity = 0.542). Adjusted for age, gender, BMI, hypertension, diabetes mellitus, PNI1, baseline NIHSS score, TOAST classification, reperfusion therapy, nutritional modality, LDL, HDL, TC, TG, and HbA1c. PNI, Prognostic Nutritional Index; mRS, modified Rankin Scale; NIHSS, National Institutes of Health Stroke Scale; TOAST, Trial of Org 10172 in Acute Stroke Treatment; LDL, low-density lipoprotein; HDL, high-density lipoprotein; TC, total cholesterol; TG, triglycerides; HbA1c, glycated hemoglobin.

Figure 2 illustrates the dose–response association between ΔPNI and the risk of poor 3-month functional outcomes using restricted cubic spline analysis. The overall association between ΔPNI and poor prognosis was statistically significant (*p* overall = 0.046). However, no evidence of nonlinearity was observed (*p* for non-linear = 0.542), indicating that the relationship was predominantly linear. As ΔPNI increased, the odds of poor outcome declined steadily, supporting the inverse association observed in the logistic regression models.

### Subgroup analyses of the association between ΔPNI and 3-month outcomes

**Figure 3:**
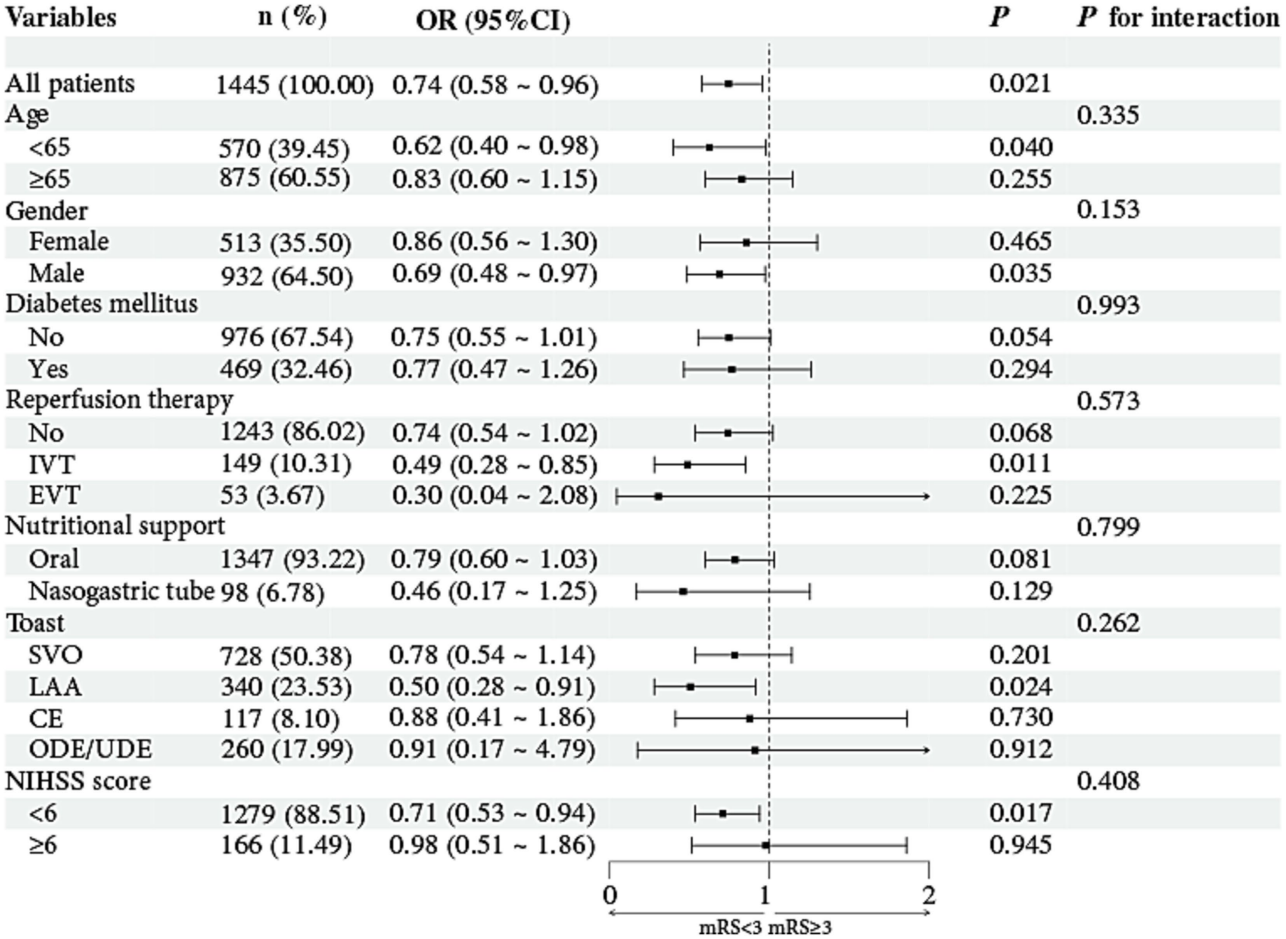
Forest plot of subgroup analyses for the association between ΔPNI and 3-month functional outcomes across key clinical strata. IVT, intravenous thrombolysis; EVT, endovascular thrombectomy; TOAST, Trial of Org 10172 in Acute Stroke Treatment; SVO, small vessel occlusion; LAA, large-artery atherosclerosis; CE, cardioembolism; ODE/UDE, other determined etiology/undetermined etiology; NIHSS, National Institutes of Health Stroke Scale.

Figure 3 shows the subgroup analyses evaluating whether the association between ΔPNI and 3-month functional outcomes varied across key clinical strata, including age, sex, diabetes mellitus, reperfusion therapy, nutritional modality, TOAST classification, and baseline NIHSS score. Higher ΔPNI was more strongly associated with a lower risk of poor outcomes in patients aged <65 years (OR: 0.62, 95% CI: 0.40–0.98, *p* = 0.040), males (OR: 0.69, 95% CI: 0.48–0.97, *p* = 0.035), those receiving IVT (OR: 0.49, 95% CI: 0.28–0.85, *p* = 0.011), patients with large-artery atherosclerosis (LAA) stroke (OR: 0.50, 95% CI: 0.28–0.91, *p* = 0.024), and those with NIHSS <6 (OR: 0.71, 95% CI: 0.53–0.94, *p* = 0.017). Nevertheless, none of the interaction terms reached statistical significance (all *p* for interaction >0.05).

### Determinants of early post-stroke nutritional status changes

**Figure 4:**
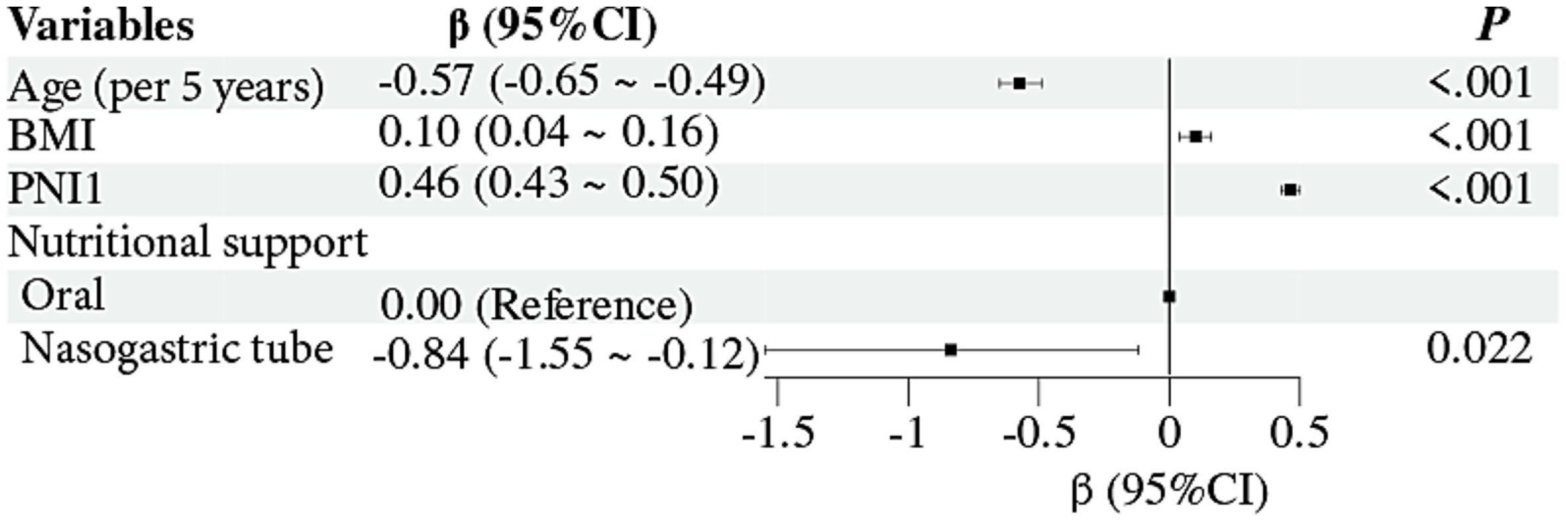
Forest plot of the multivariable linear regression analysis for factors associated with PNI change in the acute phase of ischemic stroke. BMI, body mass index; PNI, Prognostic Nutritional Index.

Figure 4 presents the results of multivariable linear regression exploring factors associated with changes in nutritional status during the acute phase of ischemic stroke. Higher baseline PNI (*β* = 0.46, 95% CI: 0.43–0.50, *p* < 0.001) and higher BMI (*β* = 0.10, 95% CI: 0.04–0.16, *p* < 0.001) were associated with improved nutritional status. In contrast, older age (per 5-year increase, *β* = −0.57, 95% CI: −0.65 to −0.49, *p* < 0.001) and nasogastric tube feeding (*β* = −0.84, 95% CI: −1.55 to −0.12, *p* = 0.022) were associated with deteriorated nutritional status.

## Discussion

This study shows that worsening nutritional status during the acute phase of ischemic stroke is associated with poorer clinical outcomes. Prior research has largely focused on baseline nutritional status, treating it merely as a static predictor of prognosis. In contrast, our findings highlight the prognostic relevance of acute-phase nutritional changes, thereby supporting the potential value of timely nutritional interventions and providing a foundation for future randomized controlled trials. In addition, we further explored patient subgroups who are more likely to benefit from improvements in nutritional status, as well as those at high risk of nutritional deterioration, offering guidance for targeted nutritional management in clinical practice.

Metabolic disorders are often accompanied by chronic low-grade inflammation, which promotes the elevation of inflammatory cytokines, leading to increased protein catabolism and decreased serum albumin levels, thereby adversely affecting nutritional indicators (25). In patients with diabetes, insulin resistance can lead to impaired glucose utilization and lipid metabolism disorders, placing the body in a state of metabolic stress that increases energy expenditure and promotes muscle protein breakdown (26). Moreover, individuals with obesity may exhibit sarcopenic obesity, a condition characterized by increased fat mass accompanied by reduced muscle mass (24). Following stroke, prolonged bed rest and reduced physical activity may further exacerbate muscle loss. Ischemic neuronal death triggers local neuroinflammation, increasing cerebral energy demand through activation of microglial glycolysis and mitochondrial oxidative phosphorylation uncoupling (27, 28). In parallel, the post-stroke stress response drives systemic metabolic mobilization by activating the neuroendocrine axis and inducing insulin resistance (29), together substantially elevating energy requirements during the acute phase of ischemic stroke. At the same time, stroke-related appetite loss, dysphagia, and impaired consciousness reduce energy intake, further placing patients at high risk of malnutrition (30). Consistently, a recent study has shown that critically ill patients with neurological injuries, including ischemic stroke, experience a more pronounced decline in nutritional status compared with those without neurological injury (31). In our study, the decline in PNI was similar on days 3, 4, and 5, supporting the pooling of these time points for analysis. This consistency suggests that nutritional deterioration may plateau within the first week, while longer-term monitoring could identify time points at which nutritional status begins to change again.

In our study, 208 patients (14.4%) had a 3-month mRS score ≥3, a proportion lower than that reported in previous studies, likely due to the inclusion of a relatively high number of patients with mild stroke. Malnutrition negatively impacts the prognosis of ischemic stroke through multiple mechanisms. Deficiencies in micronutrients, such as vitamins, exacerbate oxidative stress and vascular endothelial inflammation (32). Insufficient intake of macronutrients, particularly protein, undermines the structural and energetic foundation required for axonal regeneration, synaptic remodeling, and functional recovery (33). Malnutrition also compromises immune function, leading to a higher incidence of complications such as pneumonia, urinary tract infections, and pressure ulcers (34). Furthermore, inadequate energy intake reduces patients’ ability to engage in and tolerate rehabilitation, thereby further impeding functional recovery (35).

The decline in PNI was significantly greater in patients with poor prognosis than in those with favorable prognosis. Multivariable logistic regression identified ΔPNI as an independent predictor of ischemic stroke outcomes, with restricted cubic spline modeling further demonstrating a linear association between ΔPNI and prognosis. Greater declines in ΔPNI were associated with a significantly higher proportion of poor 3-month outcomes, and these associations remained robust after adjustment for potential confounders. When ΔPNI was categorized using different methods, patients with nutritional improvement had better outcomes than those with nutritional deterioration, while outcomes in patients with stable nutritional status did not differ significantly from either the improvement or deterioration groups. We speculate that this phenomenon may be attributable to the linear association between ΔPNI and prognosis, with patients exhibiting stable nutritional status falling within an intermediate risk range and showing relatively modest differences in outcomes. From a clinical perspective, only substantial changes in nutritional status, particularly effective improvement among patients with nutritional deterioration, are likely to result in prognostic benefits. Interestingly, PNI at admission was not associated with ischemic stroke outcomes in our study, whereas several previous studies reported a significant association. These discrepancies may be attributable to differences in study populations: some included only elderly patients (8, 9), others focused exclusively on patients undergoing reperfusion therapy (36), and some enrolled patients admitted within 1 week of stroke onset (10). Such variations in population characteristics likely account for the inconsistent findings.

Subgroup analyses indicated that changes in nutritional status had a stronger impact on the prognosis of ischemic stroke patients who were younger or middle-aged, male, diagnosed with LAA stroke, undergoing intravenous thrombolysis, or presenting with mild stroke. These observations can be explained by two complementary mechanisms. First, younger patients and those with lower baseline NIHSS scores generally have greater neuroplasticity and higher potential for functional recovery (37), within which adequate nutrition may play a more critical role in supporting neural repair and functional reorganization. Second, certain clinical contexts, such as reperfusion therapy and LAA-type ischemic stroke, are associated with increased inflammatory activity, oxidative stress, and elevated metabolic and energy demands (38, 39). In these settings, deterioration in nutritional status may be closely linked to immune dysregulation and impaired tissue repair. Additionally, sex-related differences in body composition and metabolic characteristics may further contribute to the greater susceptibility observed in male patients (40).

To explore factors influencing changes in nutritional status during hospitalization, we conducted linear regression analyses. Our findings suggest that changes in nutritional status during hospitalization for acute ischemic stroke are primarily determined by patients’ baseline nutritional reserves and the adequacy of nutritional support, rather than by stroke severity or other intrinsic stroke characteristics. Patients with limited energy reserves, such as those who are older or have low BMI, or low PNI, lack the capacity to meet the heightened energy demands induced by stroke, placing them at increased risk of nutritional deterioration. Moreover, complications associated with nasogastric tube feeding, including gastric retention, aspiration due to reflux, diarrhea (41), often prevent patients from achieving prescribed protein and caloric intake, further exacerbating nutritional decline.

This study has several limitations. First, it was conducted at a single center, potentially limiting the generalizability of the findings to other populations or healthcare settings. Second, we used PNI as the sole indicator of nutritional status; although sensitive to short-term changes, it does not fully capture all aspects of nutrition, such as micronutrient deficiencies or body composition. Third, we did not assess the actual caloric and protein intake, leading to possible inaccuracies in evaluating the true energy supply and its impact on nutritional status. Finally, post-stroke rehabilitation, as a variable potentially influencing outcomes, was not included in the analysis.

## Conclusion

In conclusion, our study demonstrates that declines in nutritional status during the acute phase of ischemic stroke are independently associated with an increased risk of poor 3-month functional outcomes, particularly in patients who are younger, male, diagnosed with LAA stroke, undergoing intravenous thrombolysis, or presenting with mild stroke. Patients with limited baseline energy reserves, older age, or those receiving nasogastric tube feeding are at higher risk of nutritional deterioration. These findings emphasize the importance of closely monitoring and actively managing nutritional status during hospitalization. Future high-quality randomized controlled trials are warranted to determine whether timely nutritional interventions can improve outcomes and provide evidence-based guidance for acute-phase nutritional management in ischemic stroke patients.

## Data Availability

The raw data supporting the conclusions of this article will be made available by the authors, without undue reservation.
